# Immunocytes Rapid Responses Post-ischemic Stroke in Peripheral Blood in Patients With Different Ages

**DOI:** 10.3389/fneur.2022.887526

**Published:** 2022-05-13

**Authors:** Haiyue Zhang, Jingwei Guan, Hangil Lee, Chuanjie Wu, Kai Dong, Zongjian Liu, Lili Cui, Haiqing Song, Yuchuan Ding, Ran Meng

**Affiliations:** ^1^Department of Neurology, Xuanwu Hospital, Capital Medical University, Beijing, China; ^2^Department of Neurosurgery, Wayne State University School of Medicine, Detroit, MI, United States; ^3^Department of Rehabilitation, Beijing Rehabilitation Hospital, Capital Medical University, Beijing, China; ^4^Advanced Center of Stroke, Beijing Institute for Brain Disorders, Beijing, China

**Keywords:** acute ischemic stroke, age, immune cells, NK cells, T cells

## Abstract

**Objectives:**

To explore the alterations in immune cell composition in peripheral blood in patients with acute ischemic stroke (AIS) based on their age group.

**Methods:**

Patients with imaging confirmed AIS were enrolled from April 2019 to January 2020 and were divided into three groups according to their ages: <55 years (group-A), 55**–**65 years (group-B), and >65 years (group-C). Blood samples were collected immediately when the patients were admitted to our ward prior to any intervention. Flow cytometry was used to analyze immune cell composition in peripheral blood.

**Results:**

A total of 41 eligible patients were included for final analysis. Among the three groups, the proportions of CD56^+^ CD16dim NK cells were least to greatest in group-B, group-A, then group-C, respectively. With increasing age, there was a decrease in the proportion of CD3^+^ T-cells (group-A vs. group-C, P = 0.016) and CD3+CD4+ T-cells (group-C vs. group-A, *P* = 0.008; group-C vs. group-B *P* = 0.026). Meanwhile, no significant differences in proportions of monocytes and B cells were observed.

**Conclusions:**

The compositions of immune cells in peripheral blood of AIS patients were distinct when divided by age groups. Differences in immune cell ratios may affect clinical outcomes and foreshadows possible need for customized treatment of AIS in different age groups.

## Introduction

Ischemic stroke is a general term for necrosis of brain tissue caused by stenosis, occlusion, or insufficiency of arteries that supply blood to the brain (e.g., carotid and vertebral arteries). It accounts for approximately 87% of all strokes. Acute ischemic stroke (AIS) is a life-threatening disease with high morbidity and mortality worldwide (1–3). The pathophysiology of AIS involves immune cell activation, inflammation, and programmed cell death; of these, immune responses play a pivotal role given their contribution to both tissue damage and repair (1, 4, 5). More specifically, during the acute stage, ischemia-induced immune response damages the brain; in the sub-acute stage, Treg cells and macrophages protect and repair the brain (6–9).

Aging is an important factor that affects immune response, thereby influencing clinical outcomes (10, 11). Although the elderly are among the most vulnerable to AIS, the influence of aging and consequent changes to immune response in AIS has not been studied well. Theoretically, as people grow older, ischemia-induced immune response may be modified and weakened, which may result in poorer outcomes. To elucidate the impact of aging on immune response to AIS, we analyzed the alterations in immune cell composition in peripheral blood of AIS patients based on their age groups. The results of this study may become references for predicting outcomes and designing customized treatment.

## Materials and Methods

The Ethics Committee of Xuanwu Hospital, Capital Medical University approved this study, and all patients signed informed consents prior to their enrollment.

### Patient Enrollment

**Figure 1:**
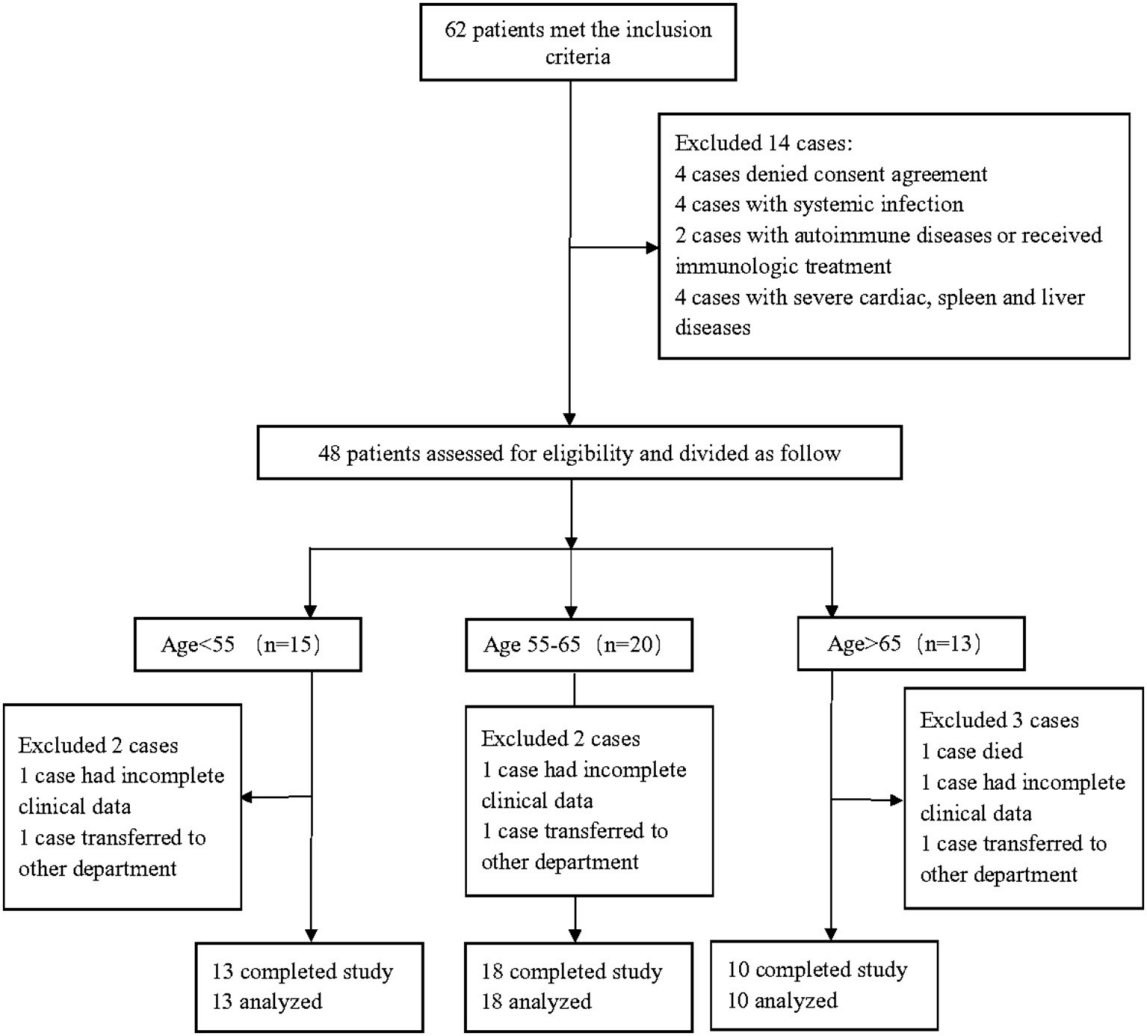
Schematic diagram of the clinical trial profile.

A total of 41 patients with imaging confirmed AIS in Xuanwu Hospital were enrolled from April 2019 to January 2020 and were divided into three groups according to their ages: <55 years (group-A), 55–65 years (group-B), and >65 years (group-C) (12, 13). Demographic data of all patients are summarized in Table 1. The flowchart of AIS patient enrollment was shown in Figure 1.

**Table 1:** Demographic data of all patients.

**Groups items**	**A (<55 years)**	**B (55–65 years)**	**C (>65 years)**	***P*** **-value**
Case number (*n*)	13	18	10	NA
Male/female (*n*)	10/3	13/5	7/3	0.927
Age (years)	43.85 ± 7.57	59.17 ± 3.11	68.1 ± 2.42	NA
Onset to door time (day)	4.54 ± 1.81	4.00 ± 1.83	3.1 ± 2.0	0.207
Smoking (*n*)	5	10	6	0.524
Stroke history (*n*)	2	2	2	0.813
Hypertension (*n*)	8	15	6	0.512
Diabetes (*n*)	4	7	6	0.354
Dyslipidemia (*n*)	5	13	5	0.158
Glycated hemoglobin(mmol/L)	6.61 ± 2.26	6.46 ± 1.55	7.03 ± 2.18	0.819
LDL (mmol/L)	2.17 ± 0.52	2.43 ± 0.94	2.31 ± 0.64	0.426
Homocysteine (umol/L)	13.43 ± 2.72	13.58 ± 3.50	14.7 ± 2.58	0.581
White blood cell (10^9^/L)	7.62 ± 1.56	7.02 ± 1.80	6.63 ± 2.50	0.461
Neutrophils (10^9^/L)	4.87 ± 1.34	4.33 ± 1.30	4.38 ± 2.57	0.662
hs-CRP(mg/ml)	1.72 ± 2.36	1.51 ± 1.47	2.36 ± 1.90	0.561

*LDL, low-density-lipoprotein; hs-CRP, hypersensitive C-reactive protein*.

Inclusion criteria included (1) age of 18–80 years; (2) stroke onset within 7 days of presentation; (3) AIS confirmed by diffusion sequence of magnetic resonance imaging (MRI-DWI); and (4) blood sample collection prior to any medication or intravenous therapy. Of note, there were no gender preferences.

Exclusion criteria included (1) systemic infection; (2) autoimmune diseases; (3) current or recent treatment with immunologic agents; (4) conditions that alter the immune system; (5) severe cardiac, spleen and liver diseases.

### Immune Cells Assay

Blood samples were collected immediately when the patients were admitted to our ward prior to any intervention. Each sample included 2 ml of fresh venous blood collected with tubes containing EDTA (Ethylene Diamine Tetraacetic Acid).

Flow cytometry (BD LSRII, Becton Dickinson, San Jose, CA, USA) was used to analyze immune cell composition in peripheral blood. Antibodies were purchased from BD Pharmingen (San Jose, CA, USA) and BioLegend (San Diego, CA, USA). Predetermined concentrations of fluorochrome-conjugated antibodies were added to 100 μl of anti-coagulated blood and incubated at room temperature for 15–20 min in the dark. Then, 2 ml of 1X Red Blood Cell (RBC) lysis solution was added to the sample and incubated again at room temperature for 10 min. After incubation, samples were centrifuged at 350xg for 5 min and supernatant was discarded. The cells in the tube were washed with at least 2 ml of Cell Staining Buffer by centrifugation at 350xg for 5 min and supernatant was discarded once again. Cells were resuspended in 0.5 ml of Cell Staining Buffer and kept shielded from light in a refrigerator set at 4–8°C.

Cells were analyzed on a BD LSRII low cytometer with FACS Diva6.1.3 software (Becton Dickinson, San Jose, CA, USA). The cells were identified as NK cells, T cells, monocytes, or B cells based on expressions of specific markers. The subcategories of cells included CD56^+^CD16dim NK-cells, CD56dimCD16^+^ NK-cells, CD16^−^CD14^+^monocytes, CD16^+^CD14^+^monocytes, CD3^+^ T-cells, CD3^+^CD4^−^CD8^−^ T-cells (double-negative T cell, DNTs), CD3^+^CD4^+^ T-cells, CD3^+^CD8^+^ T-cells, and CD19^+^ B-cells.

### Statistical Analyses

Continuous variables were represented as mean ± standard deviation (SD) or quartiles. Normality of distribution and equality of variance were assessed by the Shapiro-Wilk test and Levene's test, respectively. If the continuous variables were in normal distribution, ANOVA was used to compare the differences between the three age groups; otherwise, the Kruskal-Wallis H-test was used. LSD (least significant difference) *post-hoc* was used to compare the differences between groups. Pearson correlation coefficient and linear regression were used to predict the correlation between continuous variables. Categorical variables were analyzed with the Pearson χ2 test (GraphPad Prism 7). *P*-values < 0.05 were considered to be statistically significant.

## Results

Final analysis included data from 41 eligible patients, divided into group-A (*n* = 13), group-B (*n* = 18), and group-C (*n* = 10). Among the three groups, there were no statistically significant differences in stroke onset to door times (*P* > 0.05, Table 1).

### NK Cells Assay

**Figure 2:**
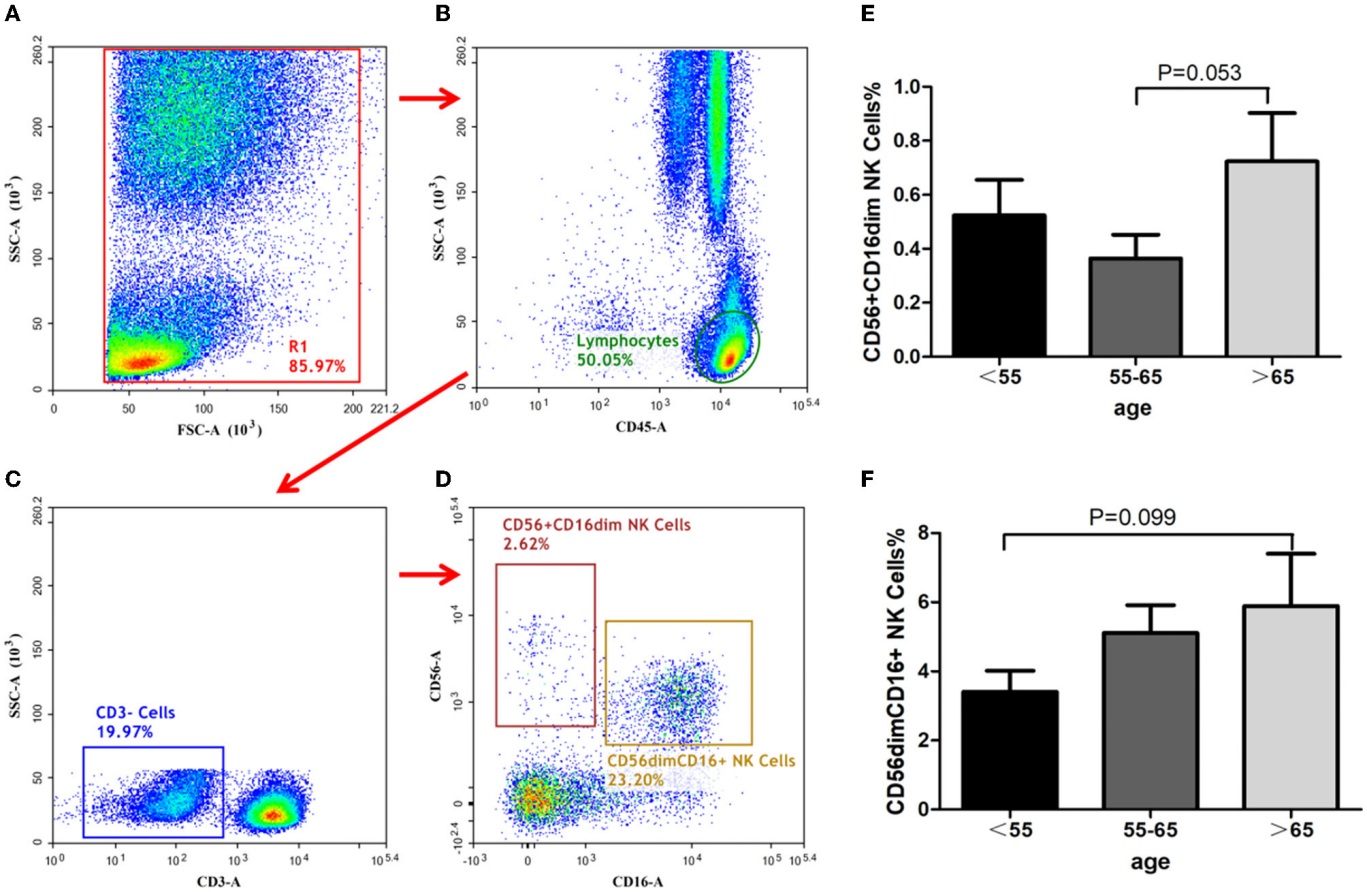
Identification of NK-cells and analysis of its subsets. NK cells identification with the gating strategy. **(A)**: (R1). **(B)**: Lymphocytes. **(C)**: CD3^−^cells. **(D)**: CD56^+^CD16dim NK cells; CD56 dim CD16^+^NK cells. Percentages of NK cell subsets among the 3 groups. **(E)**: CD56^+^CD16dim NK cells. **(F)**: CD56 dim CD16^+^NK cells.

In this study, the proportion of CD56^+^ CD16dim NK cells in peripheral blood were least to greatest in group-B, group-A, then group-C, respectively, although the differences between the groups did not reach statistical significance (Figure 2, group-B vs. group-C, *P* = 0.053). The proportion of CD56dim CD16^+^ NK cells increased with age, although it was without statistical significance (Figure 2, group-A vs. group-C, *P* = 0.099).

### Monocytes Assay

**Figure 3:**
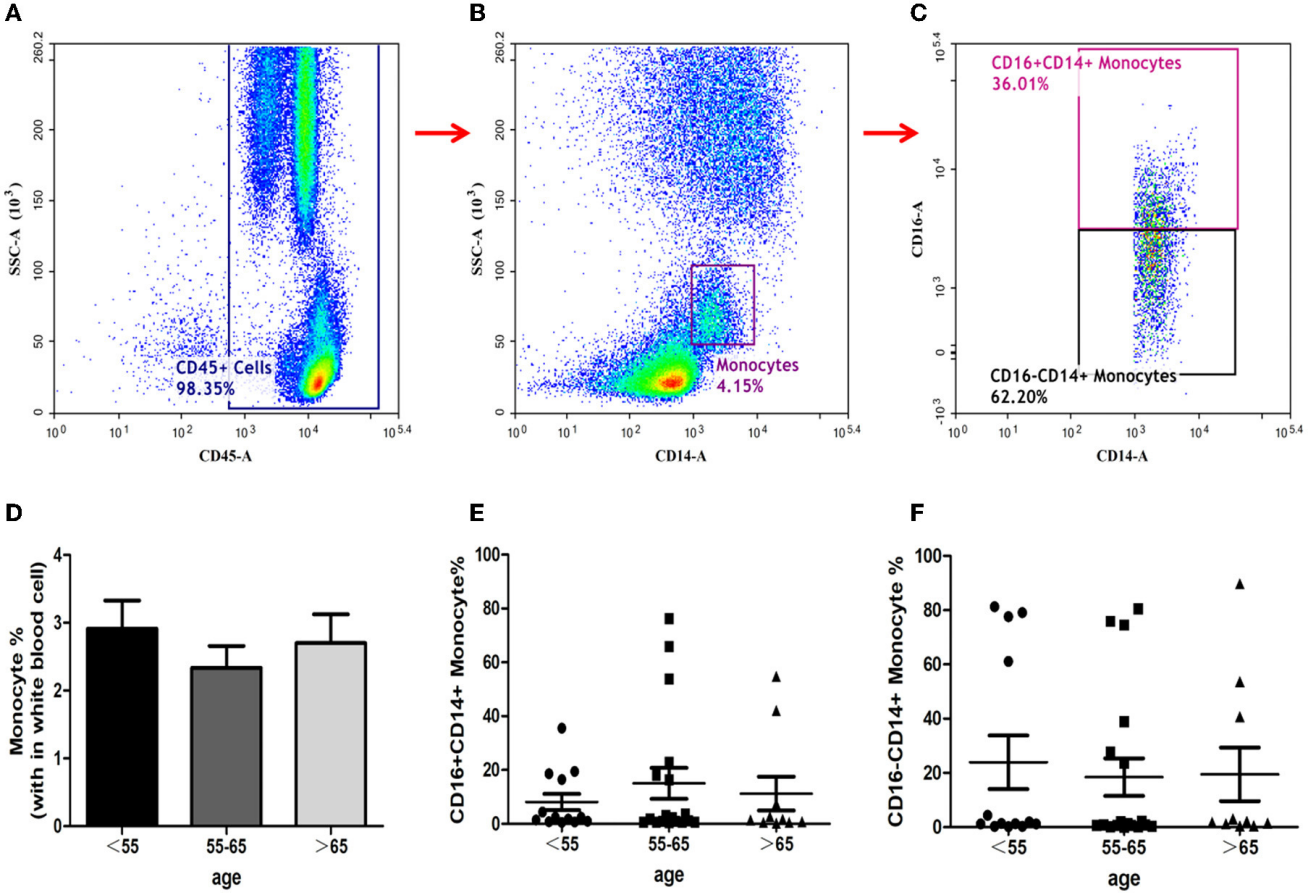
Identification of monocytes and analysis of its subsets. Monocytes identification with the gating strategy. **(A)**: CD45^+^ cells. **(B)**: Monocytes. **(C)**: CD16^+^CD14^+^ Monocytes; CD16^−^CD14^+^ Monocytes. Percentages of monocyte subsets among the 3 groups. **(D)**: Monocytes. **(E)**: CD16^+^CD14^+^ Monocytes. **(F)**: CD16^−^CD14^+^ Monocytes.

The ratios of total monocytes, CD16^−^ CD14^+^ monocytes, and CD16^+^CD14^+^ monocytes in peripheral blood have been depicted in Figures 3A–C. The proportion of total monocytes in group-B was less than those in the other two groups (Figure 3D), although the differences did not reach statistical significance. The ratios of both CD16^−^CD14^+^ and CD16^+^CD14^+^monocytes in the three groups showed no significant differences (Figures 3E,F).

### T-Cells Assay

**Figure 4:**
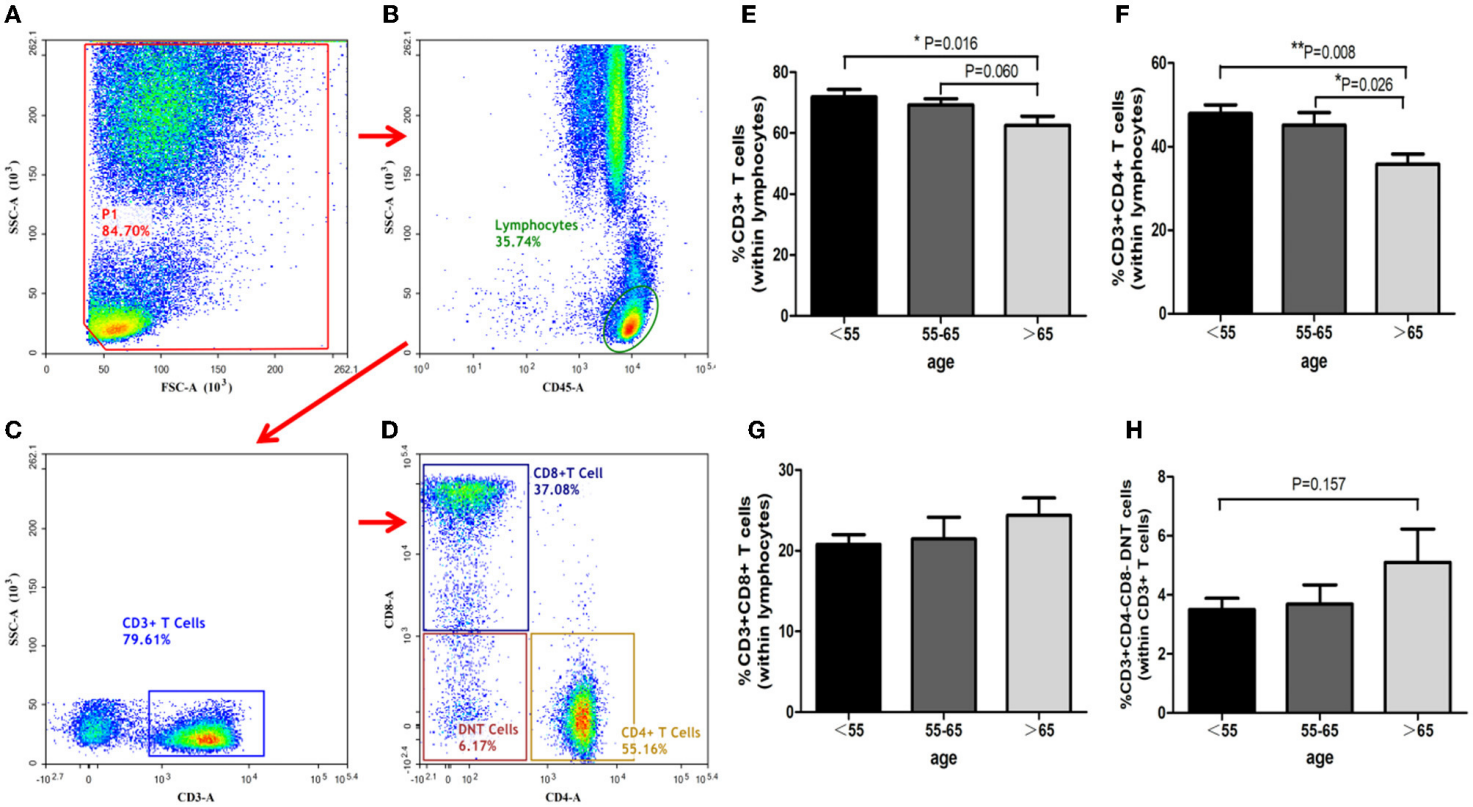
Identification of T-cells and analysis of its subsets. T cells identification with the gating strategy. **(A)**: P1. **(B)**: Lymphocytes. **(C)**: CD3^+^ T-Cells. **(D)**: CD4^+^ T-Cells; CD8^+^ T-Cells; DNT Cells. Percentages of T-Cells among the 3 groups. **(E)**: CD3^+^ T-Cells. **(F)**: CD3^+^ CD4^+^ T-Cells. **(G)**: CD3^+^CD8^+^ T-Cells. **(H)**: CD3^+^ CD4^−^ CD8^−^ T cells.

T-cells analysis showed that the proportion of CD3^+^ T-cells diminished with increasing age (ANOVA test, *P* = 0.048; group C vs. group A, *P* = 0.016; group C vs. group B, *P* = 0.060, Figure 4). The proportion of CD3^+^CD4^+^ T-cells also decreased with increasing age (group C vs. group B, *P* = 0.026; group C vs. group A, *P* = 0.008; Figure 4). However, the ratios of CD3^+^CD4^−^ CD8^−^ T-cells and CD3^+^CD8^+^ T-cells in the three groups showed no significant differences (Figure 4).

### B-Cells Assay

**Figure 5:**
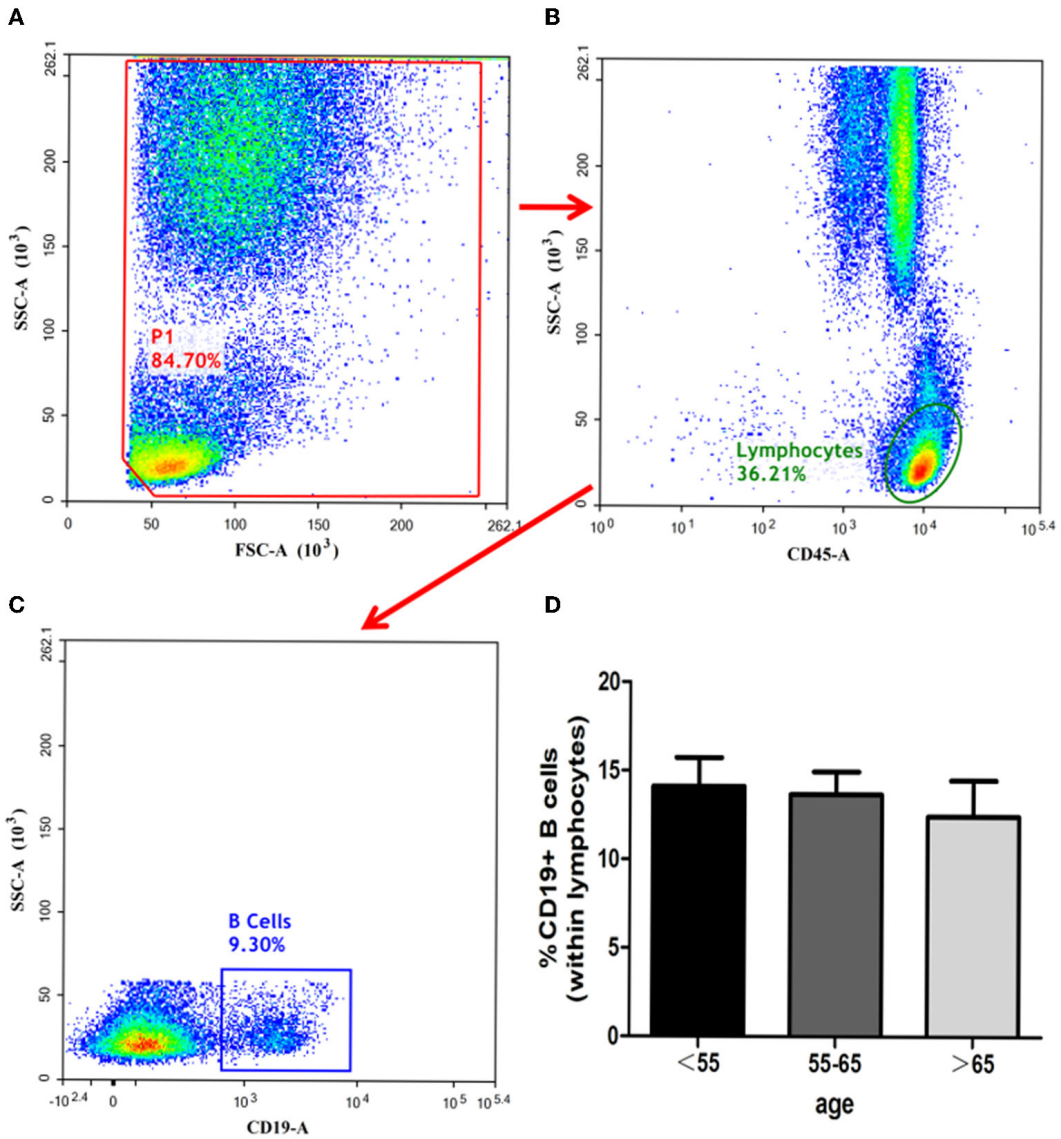
Identification of B-cells and analysis of its subsets. B-cells identified with the gating strategy. **(A)**: P1. **(B)**: Lymphocytes. **(C)**: CD19^+^ B-Cells. Percentages of B-Cells among the 3 groups. **(D)**: CD19^+^ B-Cells.

The ratios of CD19^+^ B-cells in the three groups showed no statistical significances (group C vs. group B, *P* = 0.594; group C vs. group A, P = 0.493, group B vs. group A, *P* = 0.828). The details have been depicted in Figure 5.

### Correlation Analysis Between Immune Cells and Inflammatory Biomarkers

**Figure 6:**
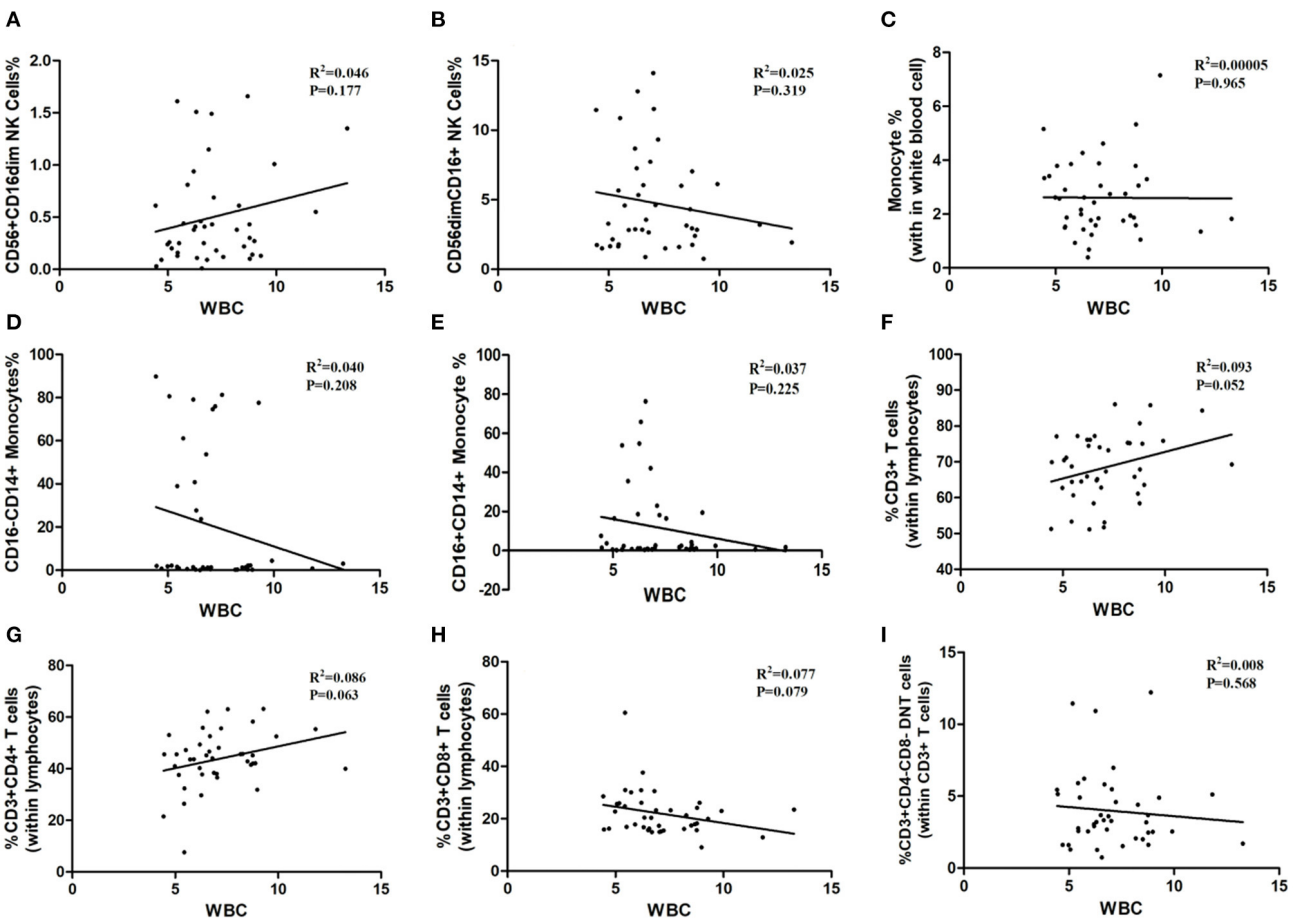
The correlation between immune cells and white blood cell. WBC: white blood cell. **(A)** CD56^+^CD16dim NK cells. **(B)** CD56 dim CD16^+^NK cells. **(C)** Monocytes. **(D)** CD16^−^CD14^+^ Monocytes. **(E)** CD16^+^CD14^+^ Monocytes. **(F)** CD3^+^ T-Cells. **(G)** CD3^+^ CD4^+^ T-Cells. **(H)** CD3^+^CD8^+^ T-Cells. **(I)** CD3^+^ CD4^−^ CD8^−^ T cells.

**Figure 7:**
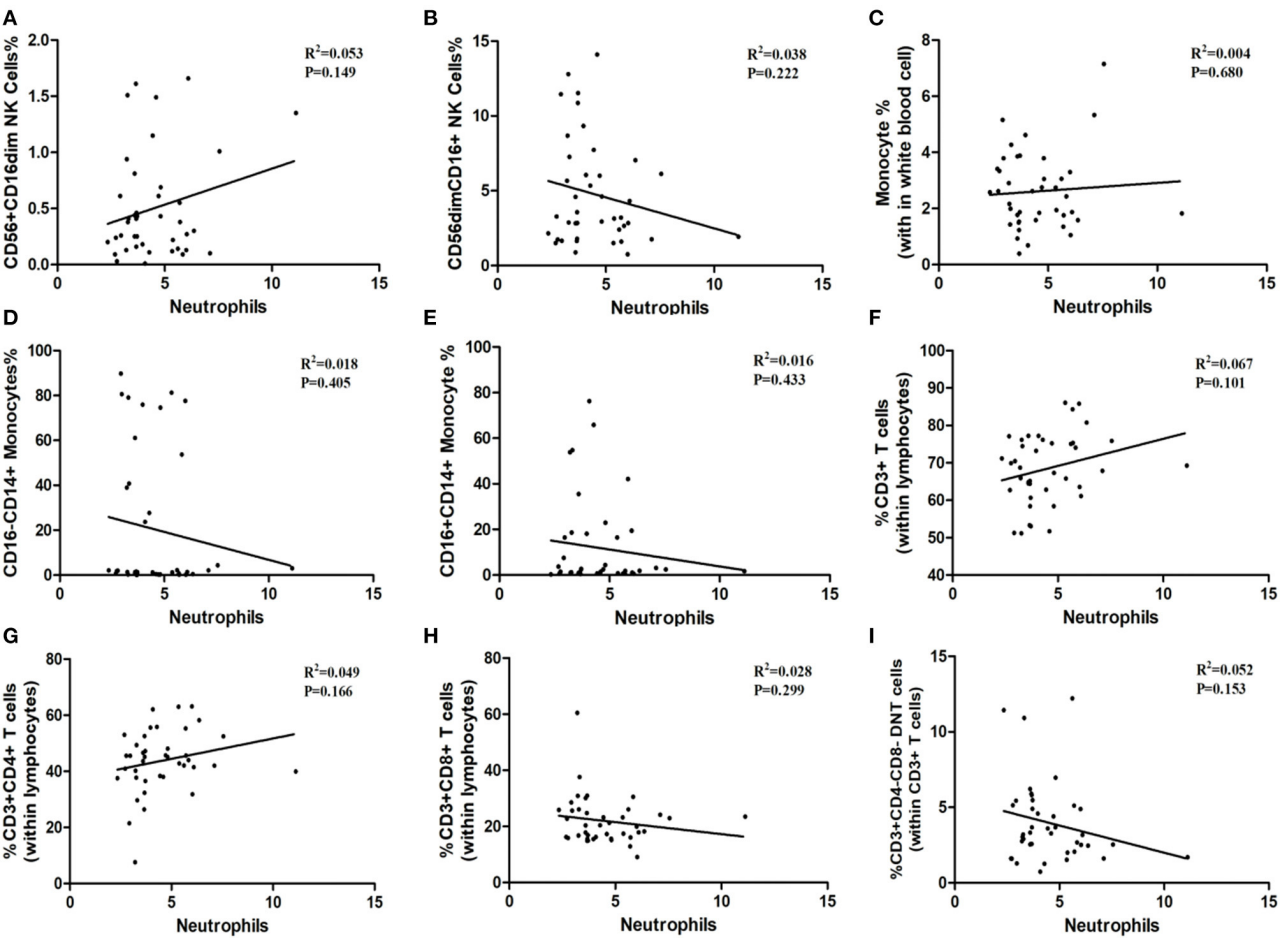
The correlation between immune cells and neutrophils. **(A)** CD56^+^CD16dim NK cells. **(B)** CD56 dim CD16^+^NK cells. **(C)** Monocytes. **(D)** CD16^−^CD14^+^ Monocytes. **(E)** CD16^+^CD14^+^ Monocytes. **(F)** CD3^+^ T-Cells. **(G)** CD3^+^ CD4^+^ T-Cells. **(H)** CD3^+^CD8^+^ T-Cells. **(I)** CD3^+^ CD4^−^ CD8^−^ T cells.

**Figure 8:**
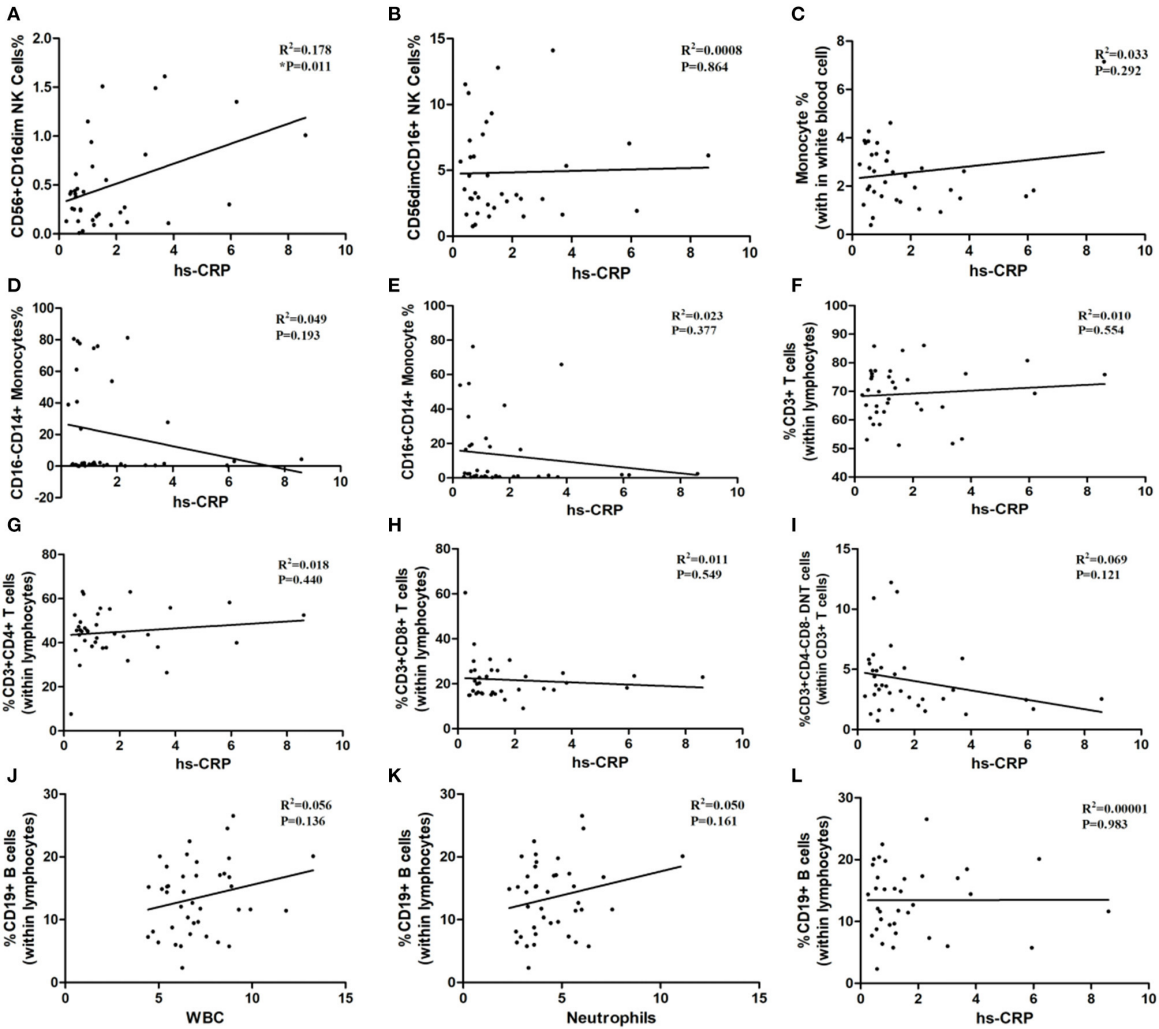
The correlation between immune cells and hypersensitive C-reaction protein and the correlation between inflammatory biomarkers and CD19^+^ B-cells. hs-CRP: hypersensitive C-reaction protein. **(A)** CD56^+^CD16dim NK cells. **(B)** CD56 dim CD16^+^NK cells. **(C)** Monocytes. **(D)** CD16^−^CD14^+^ Monocytes. **(E)** CD16^+^CD14^+^ Monocytes. **(F)** CD3^+^ T-Cells. **(G)** CD3^+^ CD4^+^ T-Cells. **(H)** CD3^+^CD8^+^ T-Cells. **(I)** CD3^+^ CD4^−^ CD8^−^ T cells. **(J–L)** the correlation between WBC, neutrophils, hs-CRP and CD19^+^ B-cells.

As shown in Table 1, the white blood cells (WBC) count, neutrophils count and hypersensitive C-reactive protein (hs-CRP) concentration in peripheral blood in three age groups showed no statistical significance. Our result indicated that the ratios of CD3^+^ T-cells and CD3^+^CD4^+^ T-cells may relate to white blood cells count (P = 0.052, P = 0.063; Figures 6F,G), although the differences did not reach statistical significance. The proportion of CD3^+^CD4^−^ CD8^−^ T-cells, CD3^+^CD8^+^ T-cells, CD56^+^ CD16dim NK cells, CD56dimCD16^+^ NK cells, total monocytes, CD16^−^CD14^+^ monocytes, and CD16^+^CD14^+^ monocytes were not correlated with white blood cells count (Figure 6). Besides, peripheral blood neutrophils count also showed no correlation with specific immune cell groups which examined in this study (Figure 7). We observed positive relevance between the percentage of CD56^+^ CD16dim NK cells and the concentration of hs-CRP (P = 0.011, Figure 8A). However, the ratios of CD19^+^ B-cells had no relevance with white blood cells (WBC) count, neutrophils count and concentration of hs-CRP (Figures 8G,K,L).

## Discussion

Immune response after AIS is a well-described phenomenon that affects clinical outcomes. It is now widely accepted that inflammation and immune mediators are potential therapeutic targets for AIS treatment due to their critical role in inducing acute neuronal damage post-AIS (1, 14). However, targeting inflammation and immune mediators are not straightforward as immune responses change with increasing age. In this study, we revealed for the first time that aging alters immune cell activity and composition in the peripheral blood of patient post-AIS. Our results offer an important reference for predicting outcomes and designing customized treatments.

During the aging process, the human immune system remodels and degenerates, which heavily impacts health outcomes (15). Both innate and adaptive immune systems' functions decline with age, which contribute to the development of age-related diseases such as neurodegenerative and cerebral vascular diseases (16, 17). The major immune cell types including monocytes, natural killer (NK) cells, dendritic cells, B lymphocytes, and T lymphocytes have been reported to play important roles in the pathogenesis of stroke (18). Since aging affects almost all kinds of immune cells, (19) we studied the alterations in immune cell composition in peripheral blood of acute ischemic stroke (AIS) patients based on their age group. The results may become a reference for future research, construction of predictive models, as well as designing of customized treatment. Previous studies have regarded AIS patients younger than 55 as young or middle-aged adults, while patients over 65 were regarded as elderly; these two populations possess distinct ischemic stroke risk factors and outcomes (12, 13, 20). In accordance to the distinct sets of risks and outcomes, our study revealed that aging affects the composition of peripheral blood immune cells.

NK cells are a vital subset of immune cells in ischemic brains. NK cells have been reported to be associated with inflammation, immunodepression, and infections post-stroke, indicating that they play multiple complex roles in stroke pathogenesis (21). Two conventional NK cell subsets, CD56^−^CD16^+^ NK-cells and CD56^+^CD16^−^ NK-cells, which are widely distributed in human peripheral blood and lymphoid tissues, were assayed in the present study. The results showed that the proportion of CD56^+^CD16^−^ NK-cells was the lowest in the age group including 55–65-year-olds, while the proportion of CD56^−^CD16^+^ NK-cells increased with aging. These results suggest that NK-cells-mediated immunosuppression and inflammation deteriorates with increasing age, for which future therapeutic strategies for the elderly should make appropriate compensations.

T-cells consist of several subsets expressing various combinations of CD3, CD4, and/or CD8 molecules, thereby functioning in diverse capacities in order to regulate immunological and inflammatory homeostasis. T-cells have been identified in brain tissue as early as 24 h after ischemia. In ischemic brain tissue, CD3^+^ T-cells were found to perform a critical role in permitting susceptibility to brain tissue injury in the early phase of ischemic stroke (22, 23). In the meantime, CD4^+^ T lymphocytes mediated the production of interleukins, tumor necrosis factor and other inflammatory factors, while CD8^+^ T lymphocytes participated in neuronal injury by releasing cytotoxic proteases (24). Both CD4^+^ and CD8^+^ T-cells aggravated brain ischemia/reperfusion injury via inflammatory and thrombogenic responses, resulting in larger infarct volumes and greater neurological deficits (25). Furthermore, CD3^+^CD4^−^CD8^−^ T-cell (double-negative T-cells, DNT) counts increased in both peripheral blood and ischemic brain tissue in patients with AIS. The infiltrating DNTs played a critical role in promoting microglia-mediated neuroinflammation and ischemic brain injury (26). In the present study, CD3+ T-cells diminished with age: The proportion of CD3^+^ T-cells in group-C (age above 65-year-old) was significantly lower than that of group-A. The CD3^+^CD4^+^ T-cells also significantly decreased with age in group-C when compared to group-A and group-B. Meanwhile, CD3^+^CD4^−^CD8^−^ T-cells and CD3^+^CD8^+^ T-cells increased with age. These results suggest that T-cell mediated immune responses in AIS were more active in younger patients when compared to the elderly population.

Monocytes present heterogeneously with proinflammatory or anti-inflammatory phenotypes depending on their stage of differentiation and the mechanism by which they were activated. Monocytes reach the central nervous system as early as 4 h from the onset of AIS. Monocytes consist of several subsets such as CD14highCD16^−^ monocytes, CD14highCD16^+^ monocytes, and CD14dimCD16^+^ monocytes; each colony of monocytes have different time courses in AIS patients and are associated with distinct clinical patterns (27). Clarifying the role of each monocyte subsets in the pathogenesis of AIS would be helpful for designing early interventions in the future. The present study found that the proportion of total monocytes nor any subset of monocytes varied significantly among the three groups. These results reveal that aging may not have significant effects on monocyte response to AIS.

B-cells are a part of the adaptive immune system and are responsible for many immunological functions. Previous studies reported that B cells did not contribute to the acute stage of stroke pathophysiology (28). Rather, B-cells were found participating in stroke recovery 4-days after stroke onset by directly protecting neurons and promoting recovery within the injured and reorganizing brain (29). The present study revealed that CD19^+^ B-cell concentrations did not vary among different age groups.

In addition, peripheral blood immune cells highly involved in brain tissue damage through stimulating inflammatory factors production in the early onset of ischemia pathological process. In order to investigate the inflammatory biomarkers' effect on the immune response whether depended on different age groups, we further analyzed the relevance between the white blood cells count, neutrophils count, hypersensitive c-reactive protein concentration with immune cells. Results showed that white blood cells count, neutrophils count and hypersensitive c-reactive protein concentration has no obvious difference between the 3 groups, thus proved the inflammatory biomarkers between the three groups has no obvious correlation with the compositions of immune cells of AIS patients. Although the percentage of CD3^+^ T cells and CD3^+^ CD4^+^ T cells may have potential correlation with the white blood cells count, but this result did not reach statistical significance obviously, still calling for further study.

## Limitations

Limitations of this study were as follows: Firstly, this was a single-center, real-world cohort study, making it susceptible to systematic errors. Secondly, the number of cases was small and without multi-timepoint analysis. The benefits of extending this study to more people and multiple timepoints did not outweigh the possible ethical concerns.

## Conclusion

The distributions of immune cells in patients with AIS in different ages were variable. The proportion of NK-cells was the lowest in patients aged 55–65 years. Ratios of both CD3^+^ T-cells and CD3^+^CD4^+^ T-cells decreased with greater age. Monocytes' and B-cells' distribution patterns did not vary significantly across the age groups, thus it may be possible that they do not greatly influence clinical outcomes of AIS. Any of the aforementioned results may impact clinical outcomes and should be considered in customized treatment.

## Data Availability Statement

The raw data supporting the conclusions of this article will be made available by the authors, without undue reservation.

## Ethics Statement

The studies involving human participants were reviewed and approved by Ethics Committee of Xuanwu Hospital, Capital Medical University. The patients/participants provided their written informed consent to participate in this study.

## Author Contributions

RM, HZ, HL, and YD drafted and revised the manuscript. RM and HZ conceptualized, designed the study, and approved the final manuscript. HZ collected, assembled, interpreted the data, and drew the figures. JG, CW, KD, ZL, LC, and HS collected the data. All authors contributed to the article and approved the submitted version.

## Funding

This work was supported by the National Key R&D Program of China under Grant 2017YFC1308400; the National Natural Science Foundation under Grants 81801143, 82171297, and 82101390; the Beijing Natural Science Foundation 7212047.

## Conflict of Interest

The authors declare that the research was conducted in the absence of any commercial or financial relationships that could be construed as a potential conflict of interest.

## Publisher's Note

All claims expressed in this article are solely those of the authors and do not necessarily represent those of their affiliated organizations, or those of the publisher, the editors and the reviewers. Any product that may be evaluated in this article, or claim that may be made by its manufacturer, is not guaranteed or endorsed by the publisher.
